# Supplementary material for: Clinicopathological Significance of RUNX1 in Non-Small Cell Lung Cancer
Source: J Clin Med. 2020 Jun 2;9(6):1694. doi: 10.3390/jcm9061694 (PMC7356912; doi:10.3390/jcm9061694)
Supplement: Supplementary file 1 [file jcm-09-01694-s001.pdf]

Journal of
Clinical Medicine

MDPI

*Supplementary Material*

# Clinicopathological Significance of *RUNX1* in Non-Small Cell Lung Cancer

**Yujin Kim [1], Bo Bin Lee [1], Dongho Kim [1], Sangwon Um [2], Eun Yoon Cho [3], Joungho Han [3], Young Mog Shim [4] and Duk-Hwan Kim [1,*]**

[1] Department of Molecular Cell Biology, Sungkyunkwan University School of Medicine, Suwon 440-746, Korea; yujin0328@hanmail.net (Y.K.), whitebini@hanmail.net (B.B.L.), jindonghao2001@hotmail.com (D.K.)
[2] Department of Internal Medicine, Samsung Medical Center, Sungkyunkwan University School of Medicine Seoul 135-710, Korea; sangwon72.um@samsung.com
[3] Department of Pathology, Samsung Medical Center, Sungkyunkwan University School of Medicine, Seoul 135-710, Korea; eunyoon.cho@samsung.com (E.Y.C.), joungho.han@samsung.com (J.H.)
[4] Department of Thoracic and Cardiovascular Surgery, Samsung Medical Center, Sungkyunkwan University, School of Medicine, Seoul 135-710, Korea
* Correspondence: dukhwan.kim@samsung.com

Received: 03 May 2020; Accepted: 27 May 2020; Published: date

**Table S1.** Prediction performance of logistic regression model based on three CpGs in a test dataset (N=269) of TCGA lung cancer.

| CpGs | AUC | SE | 95% CI[a] |
|---|---|---|---|
| cg04228935 | 0.95 | 0.02 | 0.93 - 0.98 |
| cg05000748 | 0.83 | 0.05 | 0.78 - 0.87 |
| cg11498607 | 0.92 | 0.03 | 0.89 - 0.96 |

[a] binomial exact test; Abbreviations: AUC, area under the curve; SE, standard error; CI, confidence interval.

**Table S2.** Clinicopathological characteristics.

| Variables | Value (percent) |
|---|---|
| Age | 60 ± 10 |
| Sex | |
| Male | 319 (78%) |
| Female | 90 (22%) |
| Pack-years | 29 ± 26 |
| Smoking | |
| Never | 121 (30%) |
| Former | 59 (14%) |
| Current | 229 (56%) |
| Pathologic stage | |
| I | 158 (39%) |
| II | 148 (36%) |
| III | 101 (25%) |
| IV | 2 (0.005%) |
| Histology | |
| Adenoca | 189 (46%) |
| Squamous | 192 (47%) |
| Others | 28 (7%) |
| Differentiation [a] | |

| | |
|---|---|
| Well | 65 (18%) |
| Moderately | 219 (61%) |
| Poorly | 72 (20%) |
| Undifferentiated | 5 (1%) |
| Recurrence | |
| No | 233 (57%) |
| Yes | 176 (43%) |

Abbreviations: Adenoca, adenocarcinoma; Squamous, squamous cell carcinoma [a] Differentiation data are missing for 48 patients.

© 2020 by the authors. Submitted for possible open access publication under the terms and conditions of the Creative Commons Attribution (CC BY) license (http://creativecommons.org/licenses/by/4.0/).
